# Dynamics of m6A RNA Methylome on the Hallmarks of Hepatocellular Carcinoma

**DOI:** 10.3389/fcell.2021.642443

**Published:** 2021-04-01

**Authors:** Enakshi Sivasudhan, Neil Blake, Zhi-Liang Lu, Jia Meng, Rong Rong

**Affiliations:** ^1^Department of Biological Sciences, Xi’an Jiaotong-Liverpool University, Suzhou, China; ^2^Department of Clinical Infection, Microbiology and Immunology, Institute of Infection, Veterinary and Ecological Sciences, University of Liverpool, Liverpool, United Kingdom; ^3^Institute of Integrative Biology, University of Liverpool, Liverpool, United Kingdom

**Keywords:** hepatocellular carcinoma, epitranscriptomics, m6A RNA methylation, cancer hallmarks, writers, erasers, readers

## Abstract

Epidemiological data consistently rank hepatocellular carcinoma (HCC) as one of the leading causes of cancer-related deaths worldwide, often posing severe economic burden on health care. While the molecular etiopathogenesis associated with genetic and epigenetic modifications has been extensively explored, the biological influence of the emerging field of epitranscriptomics and its associated aberrant RNA modifications on tumorigenesis is a largely unexplored territory with immense potential for discovering new therapeutic approaches. In particular, the underlying cellular mechanisms of different hallmarks of hepatocarcinogenesis that are governed by the complex dynamics of m6A RNA methylation demand further investigation. In this review, we reveal the up-to-date knowledge on the mechanistic and functional link between m6A RNA methylation and pathogenesis of HCC.

## Introduction

The advent of advancements in next-generation sequencing technologies along with the launch of highly specific antibodies capable of identifying chemically modified nucleotides broke new ground for RNA methylation, recently coined “epitranscriptomics,” to gain prominence as a dynamic and reversible modification, analogous to epigenetic regulations. While previous studies have largely focused on the genetic and epigenetic factors that contribute to hepatocellular carcinoma (HCC), research into deciphering the role of epitranscriptomics in triggering liver-related malignancies is still in its infancy. Thus, the dynamics of m6A RNA methylation on the molecular pathogenesis of HCC is an emerging field that requires extensive research, with immense potential to unlock new therapeutic targets to combat hepatocarcinogenesis.

## Hepatocellular Carcinoma

Hepatocellular carcinoma accounts for over 80% of hepatic neoplasms worldwide, imposing heavy disease burden by being the fourth most common cause of cancer-associated mortality worldwide (El-Serag and Rudolph, 2007; Fitzmaurice et al., 2019). The HCC incidence has been estimated to be more prevalent in males than females and widespread in certain regions including middle and western Africa, eastern and southern Asia, Polynesia, and Melanesia (Ferlay et al., 2010). Several risk factors contribute to HCC, among which chronic alcohol consumption, cirrhosis, viral hepatitis (due to infection with hepatitis virus B and C), non-alcoholic fatty liver disease (NAFLD), and ingestion and exposure to aflatoxin and aristolochic acid majorly contribute toward the onset of hepatocarcinogenesis (Yang and Roberts, 2010). Chronic hepatitis B and C account for the most frequent etiologies especially in particular geographical locations (such as Oceania, western sub-Saharan Africa, and central Asia) with inadequate medical resources and also the predisposition to gradual hepatic damage and ensued cirrhosis and HCC (Bosch et al., 2004; Stanaway et al., 2016). Pathologically, the regenerating nodules synthesized during cirrhosis create a favorable microenvironment for the transformation of dysplastic hepatocytes to neoplastic lesions ultimately leading to HCC (Alqahtani et al., 2019). In addition, the manifestation of HCC is seldom reported in congenital hepatic fibrosis, ataxia telangiectasia, familial cholestatic cirrhosis, familial polyposis coli, fetal alcohol syndrome, and neurofibromatosis (Leong and Leong, 2005).

Surveillance for HCC, principally in high-risk individuals, includes ultrasonography and biomarker testing (Yang and Roberts, 2010). While emerging research shows several promising biomarkers, pertaining to HCC diagnosis, prognosis, and clinical staging, such as vascular endothelial growth factor (VEGF; Biselli-Chicote et al., 2012), epidermal growth factor (EGF; Kedmi et al., 2015), platelet-derived growth factor (PDGF; Campbell et al., 2005), insulin-like growth factor (IGF; Enguita-Germán and Fortes, 2014), mammalian target of rapamycin (mTOR), and microRNAs (Mínguez and Lachenmayer, 2011; Okada et al., 2015), currently alpha-fetoprotein (AFP) is the only clinically approved serological biomarker (Beudeker and Boonstra, 2020). In fact, high expression levels of EGF and VEGF that promote proliferation and angiogenesis, respectively, have been associated with early recurrence of HCC, while TGF and PDGF receptor protein overexpression has been shown to activate profibrotic pathways that induce liver tumorigenesis (Campbell et al., 2005; Biselli-Chicote et al., 2012; Kedmi et al., 2015). Patients diagnosed with early-stage HCC opt for curative treatment options including surgical removal, orthotopic liver transplantation, or percutaneous ablation, usually performed with radiofrequency ablation or percutaneous alcohol injection. For patients with unremovable tumors, transarterial chemoembolization, carried out by infusing a concoction of chemotherapeutic agents, and transarterial radioembolization, involving treatment with radioactive particles are recommended. Besides, treatment options for patients with advanced stages of cancer include multikinase inhibitors such as sorafenib, lenvatinib, and regorafenib (Llovet et al., 2008; Yang and Roberts, 2010).

The initiation and progression of HCC are facilitated by various genetic and epigenetic alterations, which are built up in hepatocytes, eventually leading to malignant transformation as a result of the conversion of proto-oncogenes to oncogenes and the loss of functional mutation or dosage changes of tumor suppressor genes. Genetic abnormalities include chromosomal translocation, single-nucleotide polymorphisms, and targeted gene loss and deletion (Singh et al., 2018). Epigenetic changes, on the other hand, inflict no permanent genetic alterations; instead, they affect gene transcription and chromatin integrity. Epigenetic modifications that drive HCC include gene-specific DNA hypo- and hypermethylation, global genomic hypomethylation, aberrant histone modifications, and altered expression of microRNA (Leong and Leong, 2005; Libbrecht et al., 2005). Furthermore, deregulation of signal transduction pathways that govern cell cycle, proliferation, differentiation, and apoptosis, including the Wnt/β-catenin pathway, Ras/Raf/MAPK pathway, PI3/AKT/mTOR pathway, JAK/STAT pathway, and ubiquitin–proteasome pathway (UPP), can lead to onset of liver tumorigenesis (Alqahtani et al., 2019).

## Hallmarks of Hepatocellular Carcinoma

**FIGURE 1:**
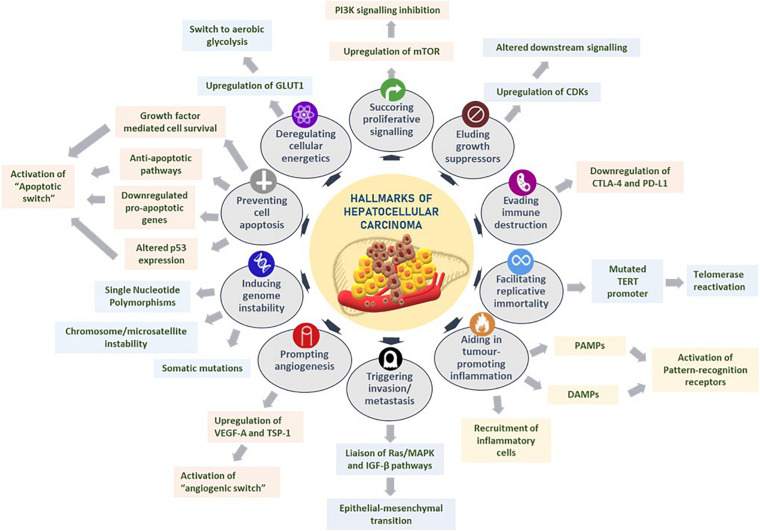
Hallmarks of hepatocellular carcinoma. Several liver tumorigenesis-driving hallmarks affect the downstream cellular mechanisms by sustaining proliferative signaling, eluding growth suppressors, evading immune destruction, facilitating replicative immortality, aiding in tumor-promoting inflammation, triggering invasion and metastasis, prompting angiogenesis, inducing genome instability, preventing cell apoptosis, and deregulating cellular energetics. Figure modified from Hanahan and Weinberg (2011).

Hanahan and Weinberg comprehensively presented the exploration of distinct and complementary traits that trigger tumorigenesis and metastatic propagation by logically organizing them into major hallmarks to rationally appreciate the complexities of neoplastic maladies (Hanahan and Weinberg, 2000, 2011). These hallmarks of cancer are as follows: sustaining proliferative signaling, eluding growth suppressors, evading immune destruction, facilitating replicative immortality, aiding in tumor-promoting inflammation, triggering invasion and metastasis, prompting angiogenesis, inducing genomic instability, preventing cell apoptosis, and deregulating cellular energetics (Hanahan and Weinberg, 2011). The hallmarks of cancer with regard to HCC are reviewed below and summarized in Figure 1.

Sustaining mitogenic signaling and evading growth suppressors in tumor cells are feasibly the most fundamental characteristics of tumor cells, unlike in normal cells that regulate cell homeostasis, especially pertaining to releasing growth-promoting signals (Hanahan and Weinberg, 2011; Sever and Brugge, 2015). For instance, HCC occurs largely as a result of uninhibited cellular proliferation resulting from a series of dysregulations in normal cell cycle regulators such as cyclin-dependent kinases (CDKs). Given the unique regenerative aptitude of hepatocytes, any reprobate cell proliferation, upregulation of CDKs, or alterations in CDK-related downstream signaling pathways and CDK inhibitors could potentiate the onset of hepatocarcinogenesis (Shen et al., 2019).

The role of the immune system in eradicating certain neoplasia and micrometastases is an area that demands further researching. Owing to the ever-alert surveillance nature of immune cells in eliminating tumor cells, it is worth exploring the potential mechanisms that solid tumors have acquired in successfully evading immunological destruction. In HCC, immune checkpoint inhibitors such as CTLA-4 and PD-L1 regulate the immunosuppression of chronic inflammation brought about by persistent expression of certain cytokines and immune cell recruitment (Makarova-Rusher et al., 2015; Xu et al., 2018). Contrary to previous beliefs that immune responses largely represented an attempt to eliminate tumorigenesis, an ever-growing assemblage of scientific evidence suggests the paradoxical effect of tumor-induced inflammation in aiding neoplasias (DeNardo et al., 2010; Qian and Pollard, 2010). HCC-associated inflammation could be chiefly attributed to recruitment of inflammatory cells in the tumor microenvironment, extrinsic pathways that activate pattern recognition receptors by pathogen-associated molecule patterns, or damage-associated molecule patterns (DAMPs) released from liver cells undergoing apoptosis (Yu et al., 2018).

Tumor cells ensure continued survival by withstanding two crucial aspects that limit unlimited replicative potential in normal cells: senescence and crisis/apoptosis. It has been observed in cirrhotic liver cells that telomerase, an enzyme that prevents telomere shortening and ensuing cellular senescence, has an impaired activity coupled with subsequent shortening of telomeres implicating senescence of hepatocytes (Nault et al., 2019). To circumvent this and enable replicative perpetuity, telomerase reactivation is elicited through aberrant mutations in TERT promoter, leading to uncontrolled cell proliferation and subsequent HCC development (Donaires et al., 2017; Nault et al., 2019).

The multistep mechanism of invasion and metastasis is broadly regulated by epithelial–mesenchymal transition (EMT), heterotypic involvement of neoplastic stromal cells, and plasticity in the invasive growth properties disseminated by cancer cells (Hugo et al., 2007; Klymkowsky and Savagner, 2009; Egeblad et al., 2010). It has been proposed that in HCC, signaling through the Ras/MAPK pathway could liaise with the TGF-β signal transduction pathway in driving the shift from EMT, rendering tumor cells their mobility (Matsuzaki et al., 2000). Another hallmark of cancer, inducing angiogenesis, involves tumor-associated neovasculature that activates an “angiogenic switch” through the regulation of countervailing inducers and inhibitors, such as VEGF-A and thrombospondin-1, respectively, Hanahan and Weinberg (2011). Given the high invasive nature of HCC, it is not surprising to observe VEGF overexpression in the precancerous stages of dysplastic and cirrhotic liver tissues further to a strong correlation of VEGF expression and tumor grading of HCC (Hamdy et al., 2020).

Acquisition of genomic instability could convene selective advantage on neoplastic cells, enabling them to outgrow and dominate in a tumor microenvironment niche. Premalignant cells drive tumorigenesis by enhancing their sensitivity to mutagenic agents and compromising the “surveillance systems” that monitor cellular genomic integrity (Jackson and Bartek, 2009). Notably in HCC, genetic alterations are instigated by chromosome and microsatellite instability, accumulated somatic mutations, single-nucleotide polymorphisms, and deregulated signaling pathways (Niu et al., 2016; Rao et al., 2017). Resisting cell death is another crucial hallmark of tumorigenesis. While programmed cell apoptosis, resulting from certain elevated oncogenic signaling mechanisms and hyperproliferation-associated DNA damage, functions as a natural barrier to carcinogenesis, certain tumors eventually progress to high-grade malignancy and induce drug resistance, through an “apoptotic switch” (Adams and Cory, 2007; Hanahan and Weinberg, 2011). Especially in HCC, apoptosis-associated mechanisms are governed by attenuation of p53 function through telomere-induced chromosomal instability (Farazi et al., 2006), downregulation of pro-apoptotic genes such as B cell lymphoma 2 (Mott and Gores, 2007), growth factor-mediated cell survival (Llovet and Bruix, 2008), and overactivation of anti-apoptotic pathways associated with fas pathway inhibitors (Lee et al., 2001).

Tumor energy metabolism, an emerging hallmark, confers the metabolic preferences of cancer cells to favor aerobic glycolysis, famously characterized as the Warburg effect (Vander Heiden et al., 2009). Gluconeogenesis in HCC is driven by upregulating facilitative glucose transporters, notably GLUT1 (Yamamoto et al., 1990), significant elevation of hypoxic regulators such as HIF-1α (Yang et al., 2014), and expression of rad and myc oncogenes that fuels glycolysis (Tiniakos et al., 1989).

Tumor pathogenesis encompasses a complex network of regulatory pathways involving various genetic, epigenetic, and epitranscriptomic mechanisms. Aberrant epitranscriptomic RNA modifications have also been shown to drive tumorigenesis due to dysregulation of RNA processing, polyadenylation, translation initiation, splicing, stability, and localization, which affect translation of tumor suppressors and oncogenes (Ferlay et al., 2010). Since each cancer type differs from the other, it is not surprising that different tumor promoters may activate different oncogenic pathways, directly or indirectly affecting RNA modifications, such as m6A and thereby their protein levels. It is evidenced that downregulation of RNA m6A modification can promote tumor progression in several types of cancers, such as glioblastoma, endometrial tumors, and leukemia (Yang and Roberts, 2010). Therefore, it is crucial to explore the relationship between aberrant RNA m6A modifications and hepatocarcinogenesis to better understand the disease etiopathogenesis.

## m6A RNA Modification and Associated Regulators

**FIGURE 2:**
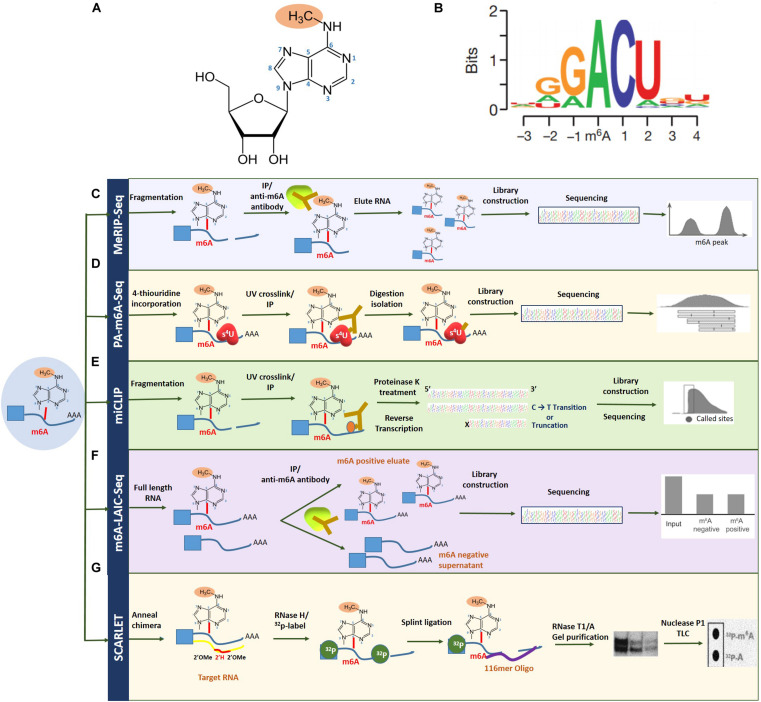
Overview of m6A RNA methylation and m6A mapping technologies. **(A)** *N*^6^-methyladenosine refers to the addition of a methyl group to the nitrogenous base present at the sixth position of the adenine residue in the RNA. **(B)** Topological analysis has indicated that m6A methylation occurs mainly near the 3′-UTR region, on adenosine residues presenting in a consensus motif of RRm6ACH (R = G/A, H = A/C/U). Transcriptome-wide sequencing technologies for mapping m6A **(C)**, MeRIP-seq **(D)**, PA-m6A-seq **(E)**, miCLIP **(F)**, and m6A-LAIC-seq **(G)** SCARLET. Figure modified from Dominissini et al. (2012); Liu et al. (2013), and Li et al. (2016).

First identified in 1970, *N*^6^-methyladenosine (m6A) is the most profuse and reversible internal modification omnipresent in eukaryotic mRNA and has been the focus in the emerging field of epitranscriptomics (Wu et al., 2016). While research has previously explored the crucial role of epigenetic regulation, pertaining to DNA and histone methylation, the dynamic function of m6A RNA modification is a relatively novel and largely unexplored territory, with regard to discovering mechanisms of gene expression regulation (Strahl and Allis, 2000; Suzuki and Bird, 2008; Wu et al., 2016). With the advent of new next-generation sequencing approaches such as MeRIP-seq, over 12,000 highly conserved methylated peaks have been identified in human and mouse transcriptomes, revealing the correlation between m6A abundance and the structural and functional aspects of a specific gene. m6A modification refers to the addition of a methyl group to the nitrogenous base present at the sixth position of the adenine residue in the RNA (Figure 2; Desrosiers et al., 1974). With an approximate estimate of 0.1–0.4% of adenosines subjected to alterations, an average of two to three m6A-modified sites have been predicted to be present in every mRNA transcript (Wei et al., 1975). Topological analysis has indicated that methylation occurs mainly near the 3′-UTR region, on adenosine residues presenting in a consensus motif of RRm6ACH (R = G/A, H = A/C/U; Figure 2; Csepany et al., 1990; Meyer Kate et al., 2012).

Owing to its dynamic and reversible nature, m6A RNA methylation is modulated by a multi-subunit complex of methyltransferase proteins known as “writers,” which add methyl groups to the adenosine; demethylases known as “erasers,” which aid in the removal of methyl groups; and RNA binding proteins known as “readers,” which bind to methylated RNA and regulate discrete downstream mechanisms (Karthiya and Khandelia, 2020).

### Writers

The methyltransferase complex is composed of several subunits with key methylases such as METTL3 and METTL14 and regulator proteins such as WTAP, METTL16, ZC3H13, and RBM15/15B (Karthiya and Khandelia, 2020). METTL3 and METTL14, which contain an *S*-adenosyl methionine (SAM) binding motif, form a core heterodimer and co-localize in nuclear speckles, with the former acting as an enzymatic component and the latter as an allosteric activator (Śledź and Jinek, 2016; Wang et al., 2016). The Wilms tumor 1 (WT1)-associated protein (WTAP), a splicing factor that modulates methylation, regulates the position of the heterodimer while indirectly increasing the catalytic capacity of methyltransferases (Ping et al., 2014; Zhang et al., 2019). RBM15/15B interacts with the methyltransferase complex with the aid of WTAP, further liaises with chromatin remodeling complexes, and modulates cortical development by recruiting the writer complex. ZC3H13 zinc finger protein, on the other hand, acts as a recruiter protein promoting localization of the writer complex in the nucleus. A relatively novel methyltransferase, METTL16, directs deposition of specific RNAs as well as U6 small nuclear RNA in addition to maintaining SAM homeostasis by adding methyl groups to the SAM synthase transcript, thereby gaining control of its stability and splicing mechanisms. Choosing a transcript for methylation is carried out by recruiting methyltransferases to specific promoters by certain transcription factors in addition to being influenced by histone modifications such as H3K36me3 and H4K20me1 (Kolasinska-Zwierz et al., 2009; Tilgner et al., 2009; Zaccara et al., 2019).

### Erasers

Eraser proteins function primarily by shaping the m6A landscape dynamically. RNA demethylases such as fat mass and obesity-associated protein (FTO; Jia et al., 2011) and AlkB family member 5 (ALKBH5; Zheng et al., 2013) belong to the ALKB family of dioxygenases that appear to have a restricted role under normal physiological conditions, with prominent functions in particular organs such as the testes and in certain ailments. Functionally, FTO performs an indirect role by sequentially oxidizing *N*^6^-methyladenosine to *N*^6^-formyladenosine, with an intermediate hydroxymethyladenosine, while ALKBH5 catalyzes direct removal of methylation (Chen and Wong, 2020).

### Readers

The fate of mRNAs containing m6A is predominantly determined by different categories of m6A-binding proteins, termed “readers,” such as YT521-B homology (YTH) domain family, heterogeneous nuclear ribonucleoproteins (hnRNPs), and IGF 2 mRNA-binding proteins (IGFBPs). Such proteins govern the m6A-related downstream cellular mechanisms in tumorigenesis, viral replication, adipogenesis, hemopoiesis, and immune regulation (Zhao et al., 2020).

The YTH domain-containing family (YTHDF) comprises YTHDF1, YTHDF2, YTHDF3, YTHDC1, and YTHDC2 proteins that recognize m6A in a methylation-dependent manner. As the first cytoplasmic reader protein to be discovered, YTHDF2 performs the function of degrading methylated RNA by directly recruiting CCR4–NOT deadenylation complex to the target transcript, inherently reducing its stability and thereafter directing bound mRNA to relevant decay sites such as processing bodies (Wang et al., 2014; Du et al., 2016). YTHDF1 protein significantly augments mRNA translation efficiency through interactions with the translation initiation factor eIF3 and in some cases in an m^7^G-cap-dependent manner (Wang et al., 2015; Liu et al., 2020a). YTHDC1 plays a pertinent role in exon selection during gene splicing (Roundtree and He, 2016). YTHDC2 acts as a putative RNA helicase that governs RNA levels during cell meiosis by forming a complex with meiosis-specific coiled-coil domain-containing protein (MEIOC; Hsu et al., 2017; Jain et al., 2018).

Heterogeneous nuclear ribonucleoprotein protein family encompasses hnRNPs such as A2/B1 (HNRNPA2B1), C (HNRNPC), and G (HNRNPG), which have affinity to structural alterations induced by m6A methylation, commonly known as “m6A switch” (Liu et al., 2015, 2017; Wu et al., 2018). HNRNPA2B1 modulates alternative splicing of mRNA transcripts and processes primary miRNAs through DGCR8-directed interactions (Alarcón et al., 2015). HNRNPC and HNRNPG impact pre-mRNA processing and pre-mRNA alternative splicing, respectively, via interactions with phosphorylated carboxy-terminal domain of enzyme RNA polymerase (Zarnack et al., 2013; Liu et al., 2017). The IGFBP family proteins use a KH RNA binding domain to identify m6A-containing transcripts and exert their function by actively recruiting RNA stabilizers such as HuR to protect mRNA transcripts from degradation (Chen and Wong, 2020).

### m6A Mapping Technologies

The field of epitranscriptomics began gaining prominence with the development of methylated RNA immunoprecipitation/m6A sequencing (MeRIP/m6A-seq), capable of conducting a site-specific analysis of m6A modification-based transcriptomic studies. This technique together with ChIP-seq depends on a specific m6A antibody to pull down m6A-containing transcripts that can subsequently be mapped by next-generation sequencing technologies (Zhang and Hamada, 2020). However, to circumvent issues with regard to identifying m6A-modified site, improved technologies such as photo-crosslinking-associated sequencing (PA-m6A-seq), which uses a photo-crosslinking method, and even single-nucleotide resolution m6A mapping are attainable (Linder et al., 2015). Furthermore, with additional UV crosslinking stratagems through techniques such as m6A individual-nucleotide-resolution crosslinking and immunoprecipitation (miCLIP), specific mutations and truncation profiles affected by the presence of m6A can be mapped precisely (Zhang et al., 2019).

Another contemporary technique for high-resolution m6A mapping is the site-specific cleavage and radioactive labeling followed by ligation-assisted extraction and thin-layer chromatography (SCARLET). As implicated by the name, the method involves site-specific cleavage and radiolabeling followed by splint ligation by DNA ligase, gel purification, and, finally, an analysis using thin-layer chromatography (Liu et al., 2013; Maity and Das, 2016). While this technique cannot employ high-throughput sequencing, newer methods such as m6A level and isoform characterization sequencing (m6A-LAIC-seq), which are compatible with high-throughput sequencing, have lately begun to see the limelight (Molinie et al., 2016). Also, since total RNA is ample without the requirement of enriching the targeted fraction of RNA, this method is deemed suitable for quantifying methylation levels in low abundance RNAs such as tRNAs (Linder et al., 2015). These m6A mapping technologies have been summarized in Figure 2. More recently, the third-generation single-nucleotide sequencing technologies such as Nanopore Direct Sequencing, capable of mapping RNA modifications at single base resolution, show promise in identifying m6A sites with improved accuracy (El-Serag and Rudolph, 2007; Fitzmaurice et al., 2019).

In recent years, several bioinformatics tools and databases addressing different purposes have been developed to organize and integrate complex datasets pertaining to m6A RNA modifications and its associated regulators. An interactive analysis of epitranscriptomic sequencing for m6A site identification can be performed by databases such as deepEA (Zhai et al., 2020) and iMRM (Liu and Chen, 2020), while RNAWRE (Nie et al., 2020), and M6A2Target (Deng et al., 2020) deposit m6A regulator datasets. Furthermore, comprehensive information on reliable m6A methylation sites and peaks from MeRIP-seq data are summarized in m6A-Atlas (Tang et al., 2020) and REPIC (Liu et al., 2020b), respectively. Bioinformatics resources such as RMDisease (Chen K. et al., 2020) and RMVar (Luo et al., 2020) can aid in better understanding the association between various epitranscriptomic modifications and their probable disease relevance.

## m6A RNA Methylation Regulators and Hallmarks of Hepatocellular Carcinoma

**FIGURE 3:**
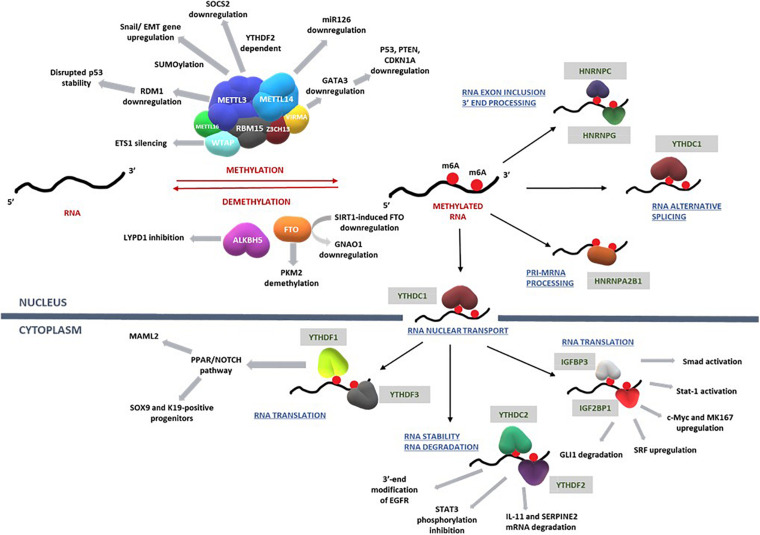
RNA methylation on transcripts induces a spectrum of intracellular mechanisms that are primarily driven by methylases, demethylases, and RNA binding proteins. Such mechanisms regulate RNA translation, nuclear transport, degradation, exon inclusion 3′-end processing, and alternative splicing. Deregulation of these complex network of mechanisms involving key signal transduction pathways that govern cell cycle, proliferation, differentiation, and apoptosis eventually influence the progression of hepatocellular carcinoma. Refer to Table 1 for information on the regulatory patterns of m6A regulators with regard to HCC. Figure modified from Karthiya and Khandelia (2020).

The impact of m6A writers, readers, and erasers on key pathways that regulate the development of HCC are summarized in Figure 3 and Table 1.

**TABLE 1:** Role of m6A methylome in hepatocarcinogenesis.

**m6A regulator type**	**m6A regulator**	**Expression pattern**	Function in hepatocellular carcinoma*	**References**
Writer	METTL3	Upregulated	METTL3 suppresses SOCS2 expression through an m6A-YTHDF2-dependent pathway.	Chen et al., 2018
Writer	METTL3	Upregulated	SUMOylation of METTL3 leads to upregulation of EMT via m6A regulation of Snail transcription factor.	Xu et al., 2020
Writer	METTL3	Upregulated	METTL3 represses the expression of RDM1, which in turn disrupts p53 protein stability.	Chen S. L. et al., 2019
Writer	METTL14	Downregulated	METTL14 liaises with microprocessor protein DGCR8 and positively modifies the microRNA 126 activity in an m6A-dependent manner.	Ma et al., 2017
Writer	WTAP	Upregulated	WTAP drives methylation of ETS1 leading to epigenetic silencing of ETS1 via a HuR-dependent manner.	Chen Y. et al., 2019
Writer	KIAA1429	Upregulated	KIAA1429, with the aid of GATA3-AS, methylates GATA3 pre-mRNA, separating HuR and degrading GATA3 pre-mRNA.	Lan et al., 2019
Eraser	FTO	Upregulated	FTO mechanistically triggers demethylation of PKM2 mRNA and enhances PKM2 translated.	Li et al., 2019
Eraser	FTO	Downregulated	Oncogenic protein SIRT1 downregulates FTO by activating RANBP2 leading to overexpression of m6A + GNAO1.	Liu et al., 2020c
Eraser	ALKBH5	Downregulated	ALKBH5 modulates post-transcriptional inhibition of LY6/PLAUR domain-containing 1 (LYPD1), which in turn is stabilized by m6A reader IGF2BP1.	Chen Y. et al., 2020
Reader	YTHDF1	Upregulated	Modulates PPAR/NOTCH signaling pathways.	Zhao et al., 2018
Reader	YTHDF2	Downregulated	YTHDF2 binds to EGFR 3′-UTR promoting degradation of EGFR mRNA.	Zhong et al., 2019
Reader	YTHDF2	Downregulated	Repressed YTHDF2 activity disrupts tumor vasculature suppression that drives IL11 and SERPINE2 mRNA decay.	Hou et al., 2019
Reader	IGFBP3	Downregulated	IGFBP-3 regulates growth suppression signals via altering TGF-β and/or Rb pathways.	Yumoto et al., 2005
Reader	IGF2BP1	Upregulated	IGF2BP1 stabilizes c-MYC and MKI67 mRNAs and enhances c-Myc and Ki-67 protein translation.	Gutschner et al., 2014
Reader	IGF2BP1	Downregulated	LINC01093 directly binds IGF2BP1, disrupting interactions between IGF2BP1 and GLI1 mRNA leading to the mRNA degradation of the latter.	He et al., 2019

*Based on *in vitro* and *in vivo* studies involving patient samples and hepatocellular carcinoma (HCC) cell lines.

### “Writers” in Hepatocellular Carcinoma Hallmarks

In a recent study, Chen et al. (2018) identified suppressor of cytokine signaling 2 (SOCS2) as a downstream target of methyltransferase METTL3-induced m6A modification via the m6A reader protein YTHDF2-dependent pathway (Chen et al., 2018). Previous studies have investigated the function of SOCS2 as a cytokine-inducible negative regulator in Janus kinase/signal transduction and activation of the JAK/STAT pathway (Yoshikawa et al., 2001). SOCS2 downregulation is significantly correlated to advanced Tumor, Node, and Metastasis staging in addition to being a prognostic marker in HCC (Qiu et al., 2013). SOCS2 has also been associated with the negative or positive regulation of GH, IGF-1, PRL, IL-2, IL-3, EPO, LIF, EGF, leptin, and IFN-α-dependent signaling pathways, which are involved in tumorigenesis (Rico-Bautista et al., 2006). Another potential regulatory mechanism involves mitogen-response SUMOylation of METTL3 that leads to upregulation of EMT genes, which inherently show a strong positive correlation to enhanced metastatic dissemination in HCC via m6A methyltransferase regulation of Snail, transcription factor, in mRNA homeostasis (Xu et al., 2020). Such mechanisms highlight the importance of m6A-mediated methylation regulation, especially on EMT, as the latter promotes drug resistance, tumor recurrence, and metastasis, all of which contribute to HCC (Wang and Zhou, 2013).

Another study explored the m6A modification of RDM1 (RAD52 motif 1) mRNA induced by the overexpression of METTL3 in clinical HCC samples. The methyltransferase represses the expression of RDM1, which in turn mechanistically disrupts protein stability of tumor suppressors such as p53 protein and subsequent suppression of Raf and ERK phosphorylation, eventually promoting tumorigenesis in HCC (Chen S. L. et al., 2019). Thus, such cascade of downstream processes leading to cancer could be solely traced back to the oncogenic behavior of METTL3.

Mechanistic studies revealed positive modulation of another subunit of methyltransferase, METTL14, on pri-miR126, a metastasis-inducing miRNA mechanism in a DGCR8-dependent manner (Ma et al., 2017). As a critical constituent of the canonical microprocessor complex for microRNA biogenesis, DGCR8 maintains the upregulation or deregulation of certain tumor-specific miRNAs that intrinsically contribute to enhanced cell proliferation, evasion of apoptosis via angiogenesis, and initiation of invasion and metastatic pathways, especially in HCC (Chu et al., 2014; Deng et al., 2019). Emerging evidence suggests that aberrantly downregulated miR126 boosted the poor overall survival associated with HCC (Bao et al., 2018). Restoring miR126 inhibited cell proliferation in HCC, arrested cell cycle advancement, and induced cell apoptosis (Zhao et al., 2015). Thus, the tumor-suppressive function of METTL14 could be a potential target in developing therapeutics targeting HCC.

Wilms tumor associated protein, another subunit of the methyltransferase complex that localizes in nuclear speckles, is highly expressed in HCC and serves as an independent predictor of hepatocarcinogenesis (Ping et al., 2014; Chen Y. et al., 2019). WTAP has been shown to promote proliferation and tumorigenesis by epigenetically silencing the ETS1 transcription factor (Chen Y. et al., 2019). Accumulating evidence suggests that ETS proteins regulate various aspects of cancer hallmarks such as proliferation inducing cell signaling, promoting angiogenesis, and evading apoptosis through enhanced nuclear transport (Myers et al., 2005), recruitment of co-repressors (Okamura et al., 2009), increased DNA binding of nuclear proteins (Sharrocks, 2001), and transactivation of certain genes such as VEGF (Tetsu and McCormick, 2017; Fry and Inoue, 2018). For example, ETS1 exhibits preferential binding to wild-type p53, suggesting tumor-suppressive functions in various cancers (Martinez, 2016). ETS1 was also shown to directly upregulate genes necessary for angiogenesis and extracellular matrix remodeling, such as the matrix metalloproteinases MMP-1, MMP-3, and MMP-9 and integrin β3 (Oda et al., 1999). Thus, WTAP-mediated epigenetic silencing of ETS1 could indicate a cogent m6A regulator-driven tumorigenesis mechanism at play.

KIAA1429, another crucial component of the m6A methyltransferase complex, is upregulated in HCC cells exhibiting poor prognosis (Lan et al., 2019). Furthermore, based on extensive *in vitro* and *in vivo* studies, they identified GATA binding protein 3 (GATA3) as a direct downstream target of KIAA1429-induced m6A modifications. GATA3 is a transcription factor composed of two zinc fingers at the carboxyl terminus, which has been linked with suppression of metastasis, tumor microenvironment modulation, and promotion of cellular differentiation (Zheng and Blobel, 2010; Chou et al., 2013; Siddiqui et al., 2014). KIAA1429, under the guidance of the long non-coding RNA (lncRNA) GATA3-AS, facilitates m6A methylation on the 3′-UTR of GATA3 pre-mRNA, disrupting the activity of the RNA binding protein HuR to GATA3 pre-mRNA, eventually downregulating GATA3 mRNA expression (Lan et al., 2019). GATA3 targets one of the classical hallmarks of cancer, the capability of tumor cells to induce invasion and metastasis (Hanahan and Weinberg, 2000; Chou et al., 2010). In fact, a previous study showed that GATA3-AS drives hepatocarcinogenesis via metastasis, particularly in HCC, by suppressing tumor suppressor genes such as p53, PTEN, and key inhibitors, namely, CDKN1A (Luo et al., 2019).

### “Erasers” in Hepatocellular Carcinoma Hallmarks

Fat mass and obesity-associated protein demethylase is significantly upregulated in HCC correlating with poor prognosis, while triggering demethylation of pyruvate kinase 2 (PKM2) mRNA (Li et al., 2019). PKM2, a rate-limiting glycolytic muscle isozyme, acts as a catalysis mediator in the irreversible transphosphorylation between adenosine diphosphate and phosphoenolpyruvate, leading to production of pyruvate and ATP (Altenberg and Greulich, 2004). In neoplastic cells, PKM2 is overexpressed under the regulation of oncoproteins such as c-Myc, which activates transcription of hnRNPs, a class of reader proteins that in turn control mRNA m6A regulation (David et al., 2010; Liu et al., 2015). PKM2 thus prompts metabolic reprogramming, a core hallmark of cancer, by facilitating anabolic metabolism in proliferating cells (Ward and Thompson, 2012). Another study that scrutinized the clinicopathological features in HCC patients with high PKM2 levels observed poor prognosis coupled with lower creatinine levels, advanced stage, and higher grade in such groups (Lu et al., 2018). Although Gene Expression Profiling Interactive Analysis (GEPIA) and The Cancer Genome Atlas (TCGA) databases show a strong correlation between the mRNA expression levels of FTO and PKM2, the exact dynamics of m6A demethylases and tumorigenesis inducing altered metabolism portrays a research gap that is yet to be filled.

Sirtuin 1 (SIRT1), also known as NAD-dependent deacetylase SIRT1, is frequently upregulated in HCC, where it regulates chemoresistance and metastasis while maintaining tumorigenicity and self-renewal capabilities of liver cancer stem cells (Wilking and Ahmad, 2015; Farcas et al., 2019). SIRT1 also upregulates oncogenes such as β-catenin (Firestein et al., 2008), HIF-1α (Laemmle et al., 2012), and c-Myc (Jang et al., 2012), especially in liver cancers. Functioning as an oncogene, SIRT1 downregulates m6A demethylase, FTO, by activating nucleoporin RaBnP2, a protein with a small ubiquitin-related modifier (SUMO) E3 ligase activity. RaBnP2 triggers SUMOylation of FTO at lysine (K)-216 site leading to FTO degradation (Liu et al., 2020c). Furthermore, guanine nucleotide-binding protein G (o) subunit alpha (GNAO1), a tumor suppressor, is an m6A-mediated downstream target of FTO. SIRT1 induces downregulation of FTO, thus leading to degradation of GNAO1, which in turn fuels hepatocarcinogenesis (Liu et al., 2020c).

AlkB family member 5 demethylase is downregulated in HCC with the functional role of suppressing proliferation and invasion capabilities of tumor cells (Chen Y. et al., 2020). Mechanistically, however, ALKBH5-regulated demethylation leads to post-transcriptional inhibition of LY6/PLAUR domain-containing 1 (LYPD1), a neurotransmitter receptor-binding protein involved in the regulation of breast and ovarian cancers, with the potential to act as a prognostic marker (Wang N. et al., 2019). Given that, in general, healthy tissues express relatively low levels of LYPD1 than most peripheral organs (Egerod et al., 2007), downregulation of ALKBH5 leads to significant upregulation of LYPD1, an established oncogenic driver of HCC (Chen Y. et al., 2020). While Chen et al. concluded that an explicit link between LYPD1 and cancer signaling pathways is yet to be established, they were able to provide evidence of the involvement of ALKBH5 in the regulation of P13K/AKT/mTOR and GTPase pathways, major hallmarks of cancer, through gene ontology and ALKBH5/LYPD1 gene knockdown studies (Etienne-Manneville and Hall, 2002; Manning and Toker, 2017; Chen Y. et al., 2020). Potential involvement of ALKBH5/LYPD1-mediated modulation could therefore explain the poor prognosis of HCC.

### “Readers” in Hepatocellular Carcinoma Hallmarks

YTH domain-containing family1 m6A reader protein is significantly upregulated in HCC. Based on gene ontology and Kyoto Encyclopedia of Genes and Genomes (KEGG) pathway analyses, YTHDF1 was found to be associated with p53, NOTCH, and peroxisome proliferator-activated receptors (PPAR) signaling pathways, which are known to aid in HCC progression (Han et al., 2019). PPAR Beta/Delta, ligand-activated transcription factors, have been known to favor pro-tumorigenicity while being central in the interplay of different cancer hallmark capabilities, such as cell proliferation, immune function, induction of angiogenesis, and senescence and replicative immortality (Wagner and Wagner, 2020). For example, in HCC cells, PPARγ activation leads to cell growth inhibition by overexpression of cell cycle arrest-inducing proteins such as cdc2, p21, p27, and CITED2, in addition to downregulation of cyclin D1, a protein that promotes cell cycle (Hsu and Chi, 2014). Contrarily, oxidative stress imposed by peroxisome proliferators and subsequent induction of PPARα enables hepatocellular proliferation while inhibiting apoptosis (Reddy et al., 1976; Tachibana et al., 2008). The NOTCH cell fate-regulatory pathway, another probable downstream target of YTHDF1, too, has been shown to be pro-oncogenic, due to its associations with NOTCH coactivator MAML2, a target of genetic alterations, and activation of Sox9- and K19-positive progenitors leading to liver tumorigenesis (Nalesnik et al., 2012; Strazzabosco and Fabris, 2012; Morell and Strazzabosco, 2014). Thus, the relationship between PPAR/NOTCH signaling pathways and the epitranscriptomic role of YTHDF1 is an area that warrants further research.

YTH domain-containing family2 is a novel regulator of tumor-promoting inflammation (Hou et al., 2019), which binds to m6A-containing RNAs and directs them to decay sites for degradation, thereby contributing toward regulating mRNA stability (Du et al., 2016). YTHDF2 inhibits STAT3 phosphorylation and tumorigenesis through interleukin 11 (IL-11) mRNA degradation, which encodes IL-6 family cytokine that triggers HCC with potential for proliferation and metastasis. The complex role of IL-11 as a pro-inflammatory cytokine in regulating immune response through activation of the JAK-STAT3 signaling pathway, in turn, provides the pro-inflammatory microenvironment required for malignant transformation and tumor progression (Bollrath et al., 2009; Kortylewski et al., 2009). In fact, another recent publication showed that IL-11 levels were significant in postsurgical HCC recurrence due to the associated enhancement of the IL-11-STAT3 signaling (Wang D. et al., 2019). Furthermore, YTHDF2 targets the mRNA degradation of serpin family E member 2 (SERPINE2), a protein present in the extracellular matrix and known to contribute to tumor invasion and metastasis through the oncogenic activation of BRAF, RAS, and MEK, which in turn influence the pro-neoplastic mechanisms of extracellular signal-regulated kinase (ERK) signaling (Bergeron et al., 2010; Yang et al., 2018).

YTH domain-containing family2 represses cell proliferation and activation of MEK and ERK in HCC cells through modification of 3′-UTR site of EGFR, a key factor in epithelial malignancies and directing the subsequent degradation of EGFR mRNA (Zhong et al., 2019). The integrative effects of an enhanced TGF-α-EGFR-MAPK activity on driving neoplasticity in HCC cells (Baek et al., 2010), coupled with a range of cancer hallmarks that EGFR influences, could potentially be negated by the upregulation of m6A regulator YTHDF2.

IGF 2 mRNA-binding protein3 is regarded as a putative tumor suppressor as well as mediator of mechanisms involving growth suppression. Yumoto et al. (2005) postulated that IGFBP3 expression in HCC was concomitant with abnormalities in the TGF-β receptor and/or effectors of its downstream signaling pathway such as Rb (Yumoto et al., 2005). It has been shown that IGFBP3 binds to its putative receptor IGFBP-3R inducing apoptosis, as well as binds to TGF-β receptor, eventually causing Smad activation and thereby inducing apoptosis. This reader protein also activates Stat-1 transcription factor and binds to nuclear receptors like RXR-α, prompting anti-apoptotic and anti-proliferative effects (Zappala et al., 2008; Shahjee and Bhattacharyya, 2014).

IGF 2 mRNA-binding proteins1 is strongly upregulated in HCC, and it stabilizes and upregulates c-Myc and MK167, two major regulators of cell proliferation and apoptosis (Gutschner et al., 2014). This oncofetal reader protein also promotes the expression of serum response factor (SRF), a critical transcription factor that regulates cell adhesion, cytoskeletal regulation, and cell migration, via m6A-mediated impairment of SRF mRNA decay (Descot et al., 2009; Leitner et al., 2011; Esnault et al., 2014). At the post-transcriptional level, IGF2BP1 controls the expression of PDLIM7 and FOXK1, two genes known to promote HCC hallmarks such as tumor proliferation and metastasis (Müller et al., 2019). Recently, another study showed that liver-specific lncRNAs directly bind IGF2BP1, enabling mRNA degradation of transcription factor glioma-associated oncogene homolog 1 (GLI1) mRNA, the latter known to be associated with the hepatocarcinogenesis-inducing Hedgehog pathway (Della Corte et al., 2017; He et al., 2019). Another contemporary work revealed that IGF2BP1 stabilizes the transcript of LINC01138, an oncogenic long intergenic non-coding RNA that promotes tumorigenesis and tumor invasion (Li et al., 2018). Thus, it can be postulated that IGF2BP1 reader proteins are involved in a variety of complex downstream mechanisms that remarkably influence metastasis hallmark of hepatocarcinogenesis, especially HCC.

## Conclusion and Future Perspectives

In recent years, immense integrative and comprehensive genomic and molecular analyses exploring potential diagnostic and prognostic targets for HCC have brought to light several prominent therapeutic solutions to curb the burden of HCC. While the role of genetic and epigenetic mechanisms on the pathogenesis of hepatocarcinogenesis has been the subject of extensive research, the network of mechanisms that governs epitranscriptomics and its associated RNA modifications, much less its association with liver tumorigenesis, is an emerging field that warrants further investigation. While the field of epitranscriptomics has witnessed a rapid upsurge in research publications in the recent decade, the mechanistic and functional aspects of m6A regulators and methylation levels in hepatocarcinogenesis, particularly HCC, still remains ambiguous, metaphorically signified by the parable of the “blind men and the elephant.” We are yet to find cogent answers to prevailing questions such as the following: How does m6A methylation affect the gene expression regulations in liver tumorigenesis? Which interwoven regulatory networks of pathways contribute toward m6A methylation and the expression of m6A regulator proteins? What m6A-associated mechanistic pathways are modified by external factors such as hepatitis B and C, aflatoxin exposure, and other causal factors of HCC? Do m6A regulators function as oncogenes or tumor suppressors? Only upon identifying the major cancer hallmark influencers of epitranscriptomic regulation can we successfully design therapeutic targets to combat HCC.

## Author Contributions

ES contributed to manuscript writing, figure preparation, and final editing. NB contributed to critically revising the article and rectifying grammatical errors. ZL, JM, and RR contributed to this manuscript with conception and revision.

## Conflict of Interest

The authors declare that the research was conducted in the absence of any commercial or financial relationships that could be construed as a potential conflict of interest.
